# Diagnostic Performance of Ultra-Low-Dose Computed Tomography for Detecting Asbestos-Related Pleuropulmonary Diseases: Prospective Study in a Screening Setting

**DOI:** 10.1371/journal.pone.0168979

**Published:** 2016-12-29

**Authors:** Marysa Schaal, François Severac, Aissam Labani, Mi-Young Jeung, Catherine Roy, Mickaël Ohana

**Affiliations:** 1 Service de Radiologie, Centre Hospitalier de Haguenau, Haguenau, France; 2 Service de Santé Publique, Hôpital Civil, Strasbourg, France; 3 Service de Radiologie, Nouvel Hôpital Civil, Strasbourg, France; 4 iCube Laboratory, Université de Strasbourg / CNRS, Illkirch, France; Northwestern University Feinberg School of Medicine, UNITED STATES

## Abstract

**Objective:**

To evaluate the diagnostic performance of Ultra-Low-Dose Chest CT (ULD CT) for the detection of any asbestos-related lesions (primary endpoint) and specific asbestos-related abnormalities, *i*.*e*. non-calcified and calcified pleural plaques, diffuse pleural thickening, asbestosis and significant lung nodules (secondary endpoints).

**Material and Methods:**

55 male patients (55.7±8.1 years old) with occupational asbestos exposure for at least 15 years and where CT screening was indicated were prospectively included. They all underwent a standard unenhanced chest CT (120kV, automated tube current modulation), considered as the reference, and an ULD CT (135kV, 10mA), both with iterative reconstruction.

Two chest radiologists independently and blindly read the examinations, following a detailed protocol. Sensitivity, specificity, positive predictive value, negative predictive value, accuracy and error rate of ULD CT were calculated using the exact method of Pearson with a confidence interval of 95%.

**Results:**

Radiation dose was 17.9±1.2mGy.cm (0.25mSv) for the ULD-CT versus 288.8 ±151mGy.cm (4mSv); *p* <2.2e^-16^.

Prevalence of abnormalities was 20%. The ULD CT’s diagnostic performance in joint reading was high for the primary endpoint (sensitivity = 90.9%, specificity = 100%, positive predictive value = 100%, negative predictive value = 97.8%), high for lung nodules, diffuse pleural thickening and calcified pleural plaques (sensitivity, specificity, PPV and NPV = 100%) and fair for asbestosis (sensitivity = 75%, specificity = 100%, PPV = 00%, NPV = 98.1%).

Intra-reader accuracy between the ULD CT and the reference CT for the primary endpoint was 98% for the senior and 100% for the junior radiologist. Inter-reader agreement for the primary endpoint was almost perfect (Cohen’s Kappa of 0.81).

**Conclusion:**

ULD CT in the screening of asbestos exposure related diseases has 90.9% sensitivity and 100% specificity, and could therefore be proposed as a first line examination.

## Introduction

The monitoring of people who have been exposed to asbestos in their professional lives relies, in most developed countries, on clinical examination, PA chest radiograph and routine pulmonary function test such as spirometry [1–3]. In France, the surveillance of workers with moderate to high occupational exposure to asbestos relies on an unenhanced chest computed tomography (CT) every 5 to 10 years, following a national consensus statement [4] that recommended the use of CT for the detection of benign or malignant pleuropulmonary diseases. In addition to an early detection of benign lesions, which in France can entitle exposed workers to financial compensation and early retirement, chest CT has also proved effective in detecting asymptomatic lung cancer [5], and is advocated as a screening tool by numerous authors in place of the PA chest radiograph [6, 7]. Like all screening tests, it is intended for asymptomatic people, and therefore must be the least deleterious possible. Minimizing the exposure to ionising radiation is therefore critical, and could be achieved through the use of an ultra-low dose (ULD) technique.

Thanks to technological development, and particularly since the implementation of iterative reconstruction techniques, it is possible to acquire a chest CT of diagnostic quality at a radiation dose as low as that of a chest x-ray from a front and lateral view: this is the ULD technique [8–13]. If several recent works dealing with the detection of pulmonary nodules by a ULD chest CT have already shown promising results [8, 14, 15], ULD’s diagnostic performance in detecting asbestos-related diseases is not yet clear, as only one study [16] has recently assessed it on 27 exposed workers in a screening setting. Confirming the diagnostic performance of ULD CT on a larger scale could establish this examination as a first-line test, consequently reducing the radiation dose delivered to this population.

The aim of this study is therefore to justify the relevance of the ULD CT in the detection of pleuropulmonary diseases related to asbestos exposure. The hypothesised theory is that the ULD CT is not inferior to the standard “full radiation dose” chest CT, with a non-inferiority threshold of 10%, in:

- the detection of global tomodensitometric abnormalities related to such an exposure (primary endpoint);
- the identification of specific pleuropulmonary abnormalities: non-calcified pleural plaques, calcified pleural plaques, diffuse pleural thickening, interstitial abnormalities suggesting asbestosis and significant lung nodules (secondary endpoints).

## Materials and Methods

Our hospital institutional review board (“*Comité de Protection des Personnes*”) approved this prospective study, and written informed consent was obtained from all participants.

### Inclusion and exclusion criteria

For a period of 10 months (July 2013 to May 2014), all patients referred to our department for chest CT screening of asbestos-related pleuropulmonary diseases were prospectively included. The only inclusion criterion was an occupational exposure to asbestos for a minimal cumulative period of 15 years. The exclusion criteria were a history of malignancy–apart from non-melanoma skin cancer–in the previous 5 years, the inability to give informed consent and the incapacity to raise the arms above the head. Height and weight were noted at the time of the examination, so as to calculate the Body Mass Index (BMI).

### CT acquisition

Each patient underwent an unenhanced chest CT with two successive helical acquisitions: one standard acquisition (120 kV, automated tube current modulation 10-300mA) used as the reference and one ULD acquisition (135 kV, 10 mA) added specifically for this study. All examinations were carried out on the same second-generation 320-row scanner (*Aquillion One Vision Edition*, Toshiba, Japan). Patients were positioned prone with their arms above their heads [17], so as to avoid gravity dependent parenchymal abnormalities in the posterior regions [18, 19]. Both examinations were acquired with a collimation of 0.5mm*80, a pitch of 0.813 and were reconstructed with Iterative Reconstruction (*AIDR-3D*, Toshiba, Japan) set in standard mode.

### CT interpretation

CT images were analysed on a dedicated workstation (*Vitrea* version 6.4, Vital Images, Minneapolis, USA), with systematic multiplanar and Maximum Intensity Projection (MIP) reconstructions. The analysis of the soft parts including the mediastinum, the intercostal space and the chest wall was made in a mediastinal window (width = 350 HU; center = 50 HU) reconstructed with a soft kernel. The analysis of the parenchyma was made using a lung window (width = 1500 HU; center = -700 HU) with a hard kernel. The window setting could be dynamically adapted by the reader.

Each examination was independently and anonymously interpreted by two chest radiologists, one senior (MO) with seven years’ experience in chest CT and one junior (MS) with four years’ experience. In a first reading session, the readers independently interpreted all the ULD images in a random order. The reference full dose acquisitions were independently interpreted three weeks later, in a different random order.

The image quality of these CT acquisitions was classified using a five-point Likert scale (1: uninterpretable examination, 2: Poor image quality, 3: Acceptable image quality, 4: Good image quality, 5: Excellent image quality). The levels 1 and 2 were considered as non-diagnostic examinations whereas the levels 3, 4 and 5 were considered as diagnostic-quality examinations.

### Qualitative CT analysis

Based on consensus studies [17, 18], we followed a detailed reading protocol focusing on pleural abnormalities, interstitial pulmonary lesions and significant nodules [20]. Non calcified and calcified pleural plaques were defined as circumscribed quadrangular pleural elevations with sharp borders and tissular or calcified density. Diffuse pleural thickening was defined as a significant (*i*.*e*. greater than 1mm) pleural thickening associated with crow's feet images, parenchymatous bands or entrapped atelectasis. Asbestosis was defined as interstitial parenchymal abnormalities (localized areas of architectural distortion with subpleural lines and/or intralobular linear opacities, traction bronchiectasis or bronchiolectasis, honeycombing) that were predominant in the peripheral region of the lower lobes. Significant lung nodules were defined as all nodules measuring at least 5mm, which were not entirely calcified and which were not fissural or strictly subpleural.

Each item within the 5 categories (non calcified and calcified pleural plaque, diffuse pleural thickening, asbestosis and significant lung nodule) was stated as present or absent in an unequivocal “yes or no” fashion.

At the end of the reading, the radiologists concluded in the presence or absence of asbestos-related global abnormalities, *i*.*e*. if the screening was positive or negative (this being the primary endpoint), and gave their diagnostic confidence using a 5-level scale (1: poor confidence in the diagnosis, *i*.*e*. not better than random guessing; 5: optimal conditions with >95% diagnostic confidence in the reading).

### Quantitative CT analysis

One radiologist (MS) measured the mean noise as the HU standard deviation of the air. A circular region of interest averaging at least 5 mm^2^ was placed within the trachea on the lung kernel images, and the measurements were repeated three times and averaged.

The dose metric recorded for both acquisitions was the dose-length product (DLP), expressed in milligray.cm (mGy.cm). The effective dose, expressed in mSv, was secondarily calculated using the following formula: Effective dose = DLP x 0.014 [21].

In total, the following points were obtained for each CT acquisition:

- The objective (mean noise) and subjective (5 levels rating) evaluation of image quality;
- The radiation dose expressed in mGy.cm;
- The final conclusion regarding the presence or absence of asbestos-related lesions (primary endpoint), and the diagnostic confidence of the reader on a 5 levels scale;
- The presence or absence of non calcified pleural plaque, calcified pleural plaque, diffuse pleural thickening, asbestosis and significant lung nodule (secondary endpoints).

All discrepant cases, for the full dose as well as the ULD acquisitions, were secondarily reviewed in a joint analysis to reach a definitive consensus.

All the results were recorded in an anonymous spreadsheet (Excel 2010, Microsoft, Seattle, USA).

### Statistical analysis

The diagnostic performance of the ULD CT for primary and secondary endpoints in a joint analysis was compared to the gold standard, represented by the final joint reading of the full dose chest CT. The sensitivity (Se), specificity (Sp), positive predictive value (PPV), negative predictive value (NPV), accuracy and error rate were calculated using a confidence interval of 95%, according to the exact method of Pearson.

The intra-reader agreement was evaluated by comparing the reading of the ULD CT to the reading of the full dose CT for each of the two readers.

The inter-reader agreement was analysed using the Cohen’s Kappa test. The confidence intervals for Cohen's Kappa coefficients were calculated using the Bootstrap method [22].

The Gaussian nature of the quantitative variables was assessed using the Shapiro-Wilk test. The radiation exposure, image quality, noise and diagnostic confidence were compared with a Student’s *t*-test when the parametric conditions were met, and otherwise with a Wilcoxon test. A *p* smaller than 0.05 was considered significant.

## Results

### Population

61 patients were referred to our department for CT screening of asbestos occupational exposure during the time of the study. 4 patients refused to participate and 2 patients couldn’t lay prone with the arms above the head due to shoulder pain. 55 patients were therefore ultimately included, all of which were male. They were aged (mean ± standard deviation) 55.7 ±8 years (minimum 44 and maximum 82). BMI was 27.4 ±4.3 (minimum 19 and maximum 32) Asbestos cumulative exposure duration was 19.6 ±5.5 years, with a minimal interval period of 12 years between the end of the exposure and the screening examination. 49% of participants were active smokers, with a mean exposure of 32.7 ±11 pack years.

### Image quality

There was an expected significant decrease in both the objective and subjective image quality to the expense of the ULD acquisition (Table 1): qualitative rating was 3.98±0.46 for the ULD CT versus 4.83±0.28 for the reference CT (*p*<0.001); image noise was 63±9 HU versus 48±9 HU (*p*<0.001).

**Table 1:** Subjective and objective image quality evaluation

	ULD Acquisition	Full Dose Acquisition	*p*
**Subjective image quality**			
**Average**	3.98	4.83	*p* <0.001(Wilcoxon test)
**Standard deviation**	0.46	0.28	
**Median**	4	5	
**Q1**	3.5	4.5	
**Q3**	4.5	5.0	
**Objective Image Noise**			
**Average**	62.57	48.16	*p* < 0.001(Student t test with equal variance)
**Standard deviation**	9.34	9.32	
**Median**	62.45	49.35	

**Fig 1:**
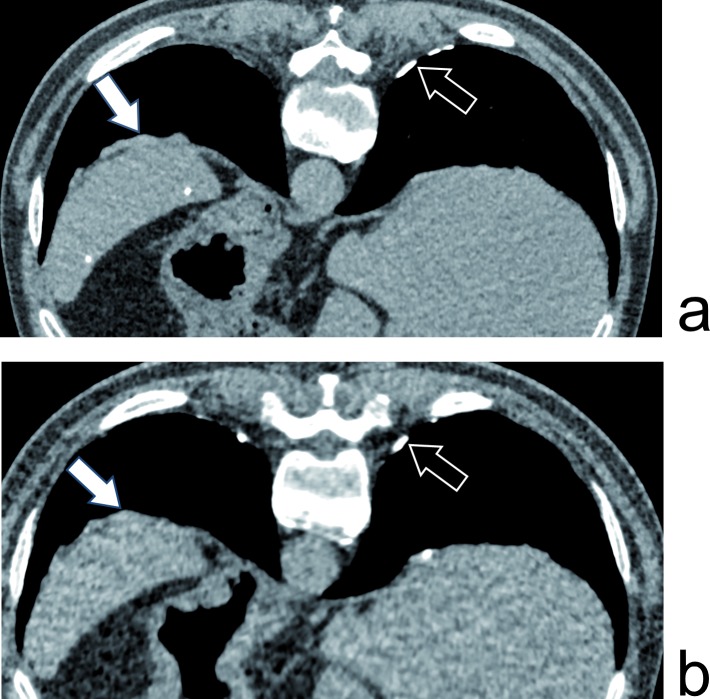
Pleural plaques. 61yo male with 19 years of asbestos exposure. Pleural plaques, calcified (black arrow) and non-calcified (white arrow) are depicted with equal efficiency by the reference full dose CT (a–DLP of 301.7mGy.cm) and the ULD acquisition (b–DLP of 19mGy.cm).

**Fig 2:**
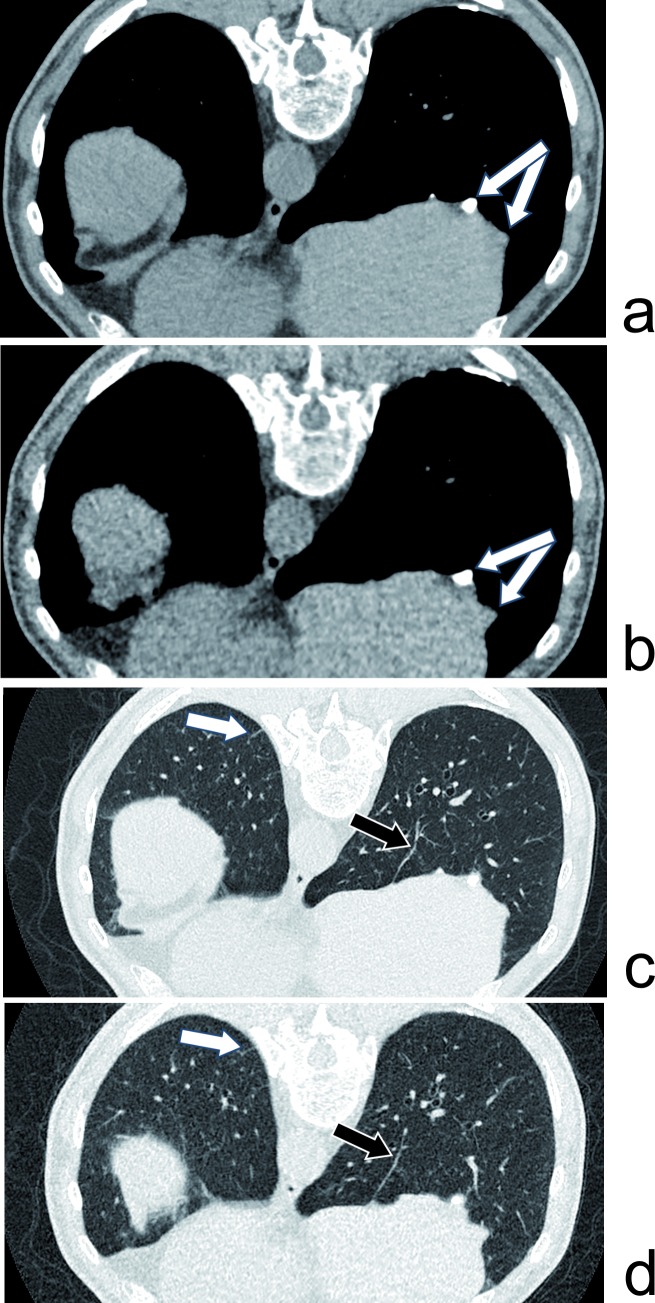
Asbestosis. 53yo male with 23 years of asbestos exposure. Pleural plaques (long white arrows), diffuse pleural thickening (parenchymal bands–black arrows) and asbestosis (subpleural intralobular and septal lines–white arrows) are depicted with equivalent diagnostic quality in the standard acquisition (a–mediastinal window, c–lung window; DLP of 291mGy.cm) and in the ULD CT (b–mediastinal window, d–lung window; DLP of 18mGy.cm).

**Fig 3:**
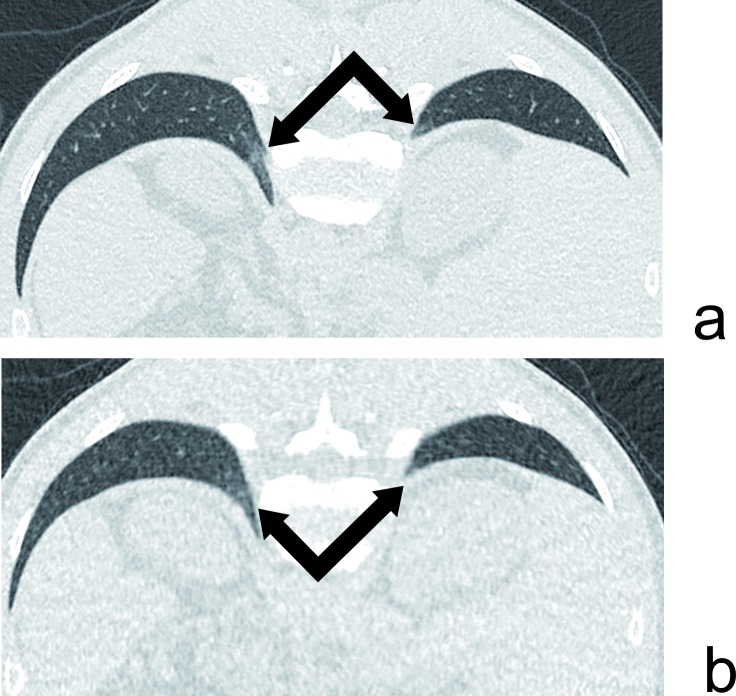
ULD CT false negative example. 47yo male with 15 years of asbestos exposure. Subtle posterior basal interstitial abnormalities related to early asbestosis are visible in the standard acquisition (a–DLP of 201mGy.cm) but indiscernible on the ULD CT (b–DLP of 19mGy.cm).

**Fig 4:**
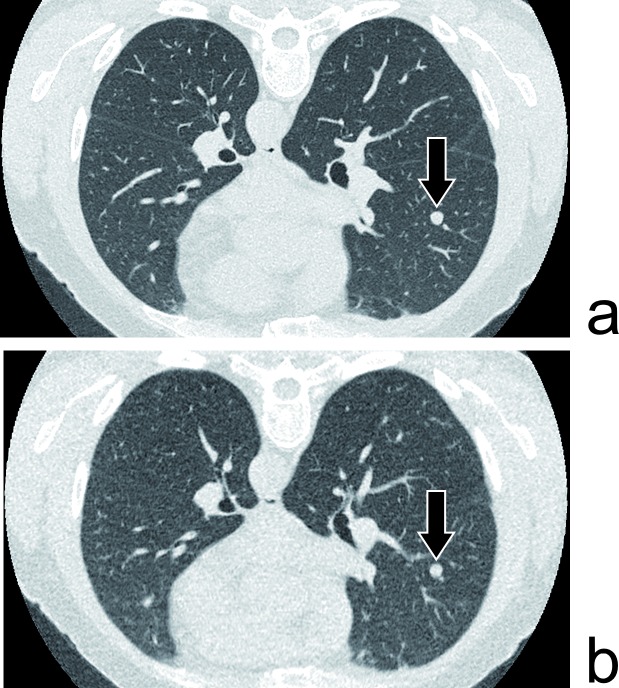
Nodule. 59yo male with 17 years of asbestos exposure. An 8mm solid nodule is depicted as well in the reference full dose acquisition (a–DLP of 475mGy.cm) as in the ULD one (b–DLP of 19mGy.cm).

Examples are shown in Figs 1–4.

### Evaluation of radiation exposure

The radiation exposure was logically significantly decreased in the ULD CT, with a radiation dose divided by 16 (*p* ≤ 2.2e^-16^, Wilcoxon test).

The average DLP was 17.9 mGy.cm (effective dose of 0.25 mSv) for the ULD CT (Standard Deviation = 1.2 mGy.cm, Median = 18 mGy.cm, Q1 = 17.2 mGy.cm, Q3 = 18.9 mGy.cm) versus 288.8 mGy.cm (effective dose of 4 mSv) for the standard full dose acquisition (Standard Deviation = 151.3 mGy.cm, Median = 242.5 mGy.cm, Q1 = 183.4 mGy.cm, Q3 = 345.5).

### Diagnostic performance of the ULD CT

The prevalence of global abnormalities was 20% (11 patients out of 55).

Results from the joint analysis on the diagnostic performance of the ULD CT for the primary and secondary endpoints are detailed in S1 Table. Out of the 55 patients, there were 44 true negatives, 10 true positives, 1 false negative and no false positive, resulting in a Sensitivity of 91% and a Specificity of 100%. The non-inferiority of the ULD CT in the detection of global pleuropulmonary abnormalities related to asbestos is thus confirmed, with a 10% margin error.

When comparing one reader’s interpretation of the ULD acquisition with his own interpretation of the reference full dose acquisition (details in S2 Table), the intra-reader agreement for the primary endpoint was 98.2% for the senior reader and 100% for the junior reader, demonstrating low intra-reader variability induced by the use of a ULD technique. This was achieved with the same diagnostic confidence between the ULD and the full dose acquisitions, as no significant difference was demonstrated between both (*p* = 0.6075, Wilcoxon signed rank test with continuity correction data). Indeed, the average diagnostic confidence of the two readers was 4.55 out of 5 for the ULD acquisition (Standard Deviation = 0.48, Median = 4.5, Q1 = 4.25, Q3 = 5.0) and 4.52 for the standard acquisition (Standard Deviation = 0.56, Median = 4.5, Q1 = 4.25, Q3 = 5.0) (p = 0.6075, Wilcoxon test).

The inter-reader agreement ranged from substantial to almost perfect, according to Cohen’s Kappa (Table 2).

**Table 2:** Inter-reader agreement.

	FULL DOSE ACQUISITION			ULD ACQUISITION		
	Cohen's Kappa		*p*	Cohen's Kappa		*p*
	Value	Confidence interval		Value	Confidence interval	
**Global abnormalities**	**0,756**	0,545–0,936	1,61 e ^-8^	**0,810**	0,624–1,000	9,39 e ^-10^
**Non calcified pleural plaques**	**0,787**	0,574–0,948	5,37 e ^-9^	**0,887**	0,744–1,000	3,64 e ^-11^
**Calcified pleural plaques**	**1**	Cannot be estimated because perfect agreement on the sample	1,21 e ^-13^	**1**	Cannot be estimated because perfect agreement on the sample	1,21 e ^-13^
**Diffuse pleural thickening**	**0,650**	0,411–1,000	3,48 e ^-7^	**0,543**	- 0,028–1,000	3,48 e ^-7^
**Asbestosis**	**0,732**	0,312–1,000	1.77e ^-8^	**0,791**	0,000–1,000	2,00 e ^-9^
**Significant lung nodules**	**0,791**	0,548–1,000	2,00 e ^-9^	**0,791**	0,548–1,000	2,00 e ^-9^

## Discussion

This study consolidates the diagnostic performance of the ULD CT in the detection of abnormalities related to asbestos exposure, with a radiation dose 16 times lower than a standard CT acquisition. For the primary endpoint which was the detection of global abnormalities related to asbestos, the ULD had a specificity and a PPV of 100%, a NPV of 97.8% and a sensitivity of 90.9% due to one single false negative (*Fig 3*). These results are in line with those of Tekath et al. [16] and confirm the feasibility and the performance of ULD in this indication on a larger population. Moreover, our study is based on standard iterative reconstructions techniques which are readily available on the vast majority of CT installed base, while the more advanced Model-Based Iterative Reconstruction technique used by Tekath et al. has limited availability and require an additional 20 to 40 minutes of computational time. Consequently, our results could be easier to translate to routine workflow.

There was an almost-perfect inter-reader agreement on the ULD acquisition (Kappa = 0.810), which demonstrates reproducibility in the analysis of the ULD acquisitions. The inter-reader agreement was also substantial in the reference full dose acquisition (Kappa = 0.756). This is largely due to the use of a detailed standardised reading protocol [17, 18].

Regarding the secondary endpoints, the calcified pleural plaques are the abnormalities easiest to recognise; they were constantly detected by both readers *(*Figs 1 and 2*)*.

The ULD CT also performed well for the detection of solid nodules greater than 5 mm, constantly detected by both readers (Fig 4). This is on par with what has already been described in other studies dedicated to nodule imaging with ULD CT [8, 11, 14, 15].

The interstitial abnormalities suggesting asbestosis remained the most difficult to detect, as the sensitivity of the ULD CT was only 75% (3 cases out of only 4). There was indeed one false negative where subtle subpleural interlobular reticulations remained undetected on the ULD by both readers (Fig 3). These abnormalities had been identified by the senior reader in the standard protocol, but not by the junior one, reflecting a challenging diagnosis. These results are in agreement with the literature, which highlights the limitations of the ULD CT in the detection of interstitial abnormalities and limited ground glass opacities [15].

Even though the noise was increased by 30% in the ULD acquisition and the subjective image quality decreased by 18%, the diagnostic confidence was not altered, both for the junior as well as the senior reader. This showed that the ULD acquisition contained sufficient information to make a comfortable diagnosis, with as higher confidence as the full dose CT.

When considering radioprotection, the necessity to reduce the diagnostic dose is all the more important for screening tests as it concerns asymptomatic patients. Carcinogenesis being a stochastic effect of ionising radiation, the screening of diseases related to asbestos exposure must be the least radiating possible, even more so since we are dealing with people with a higher risk of lung cancer. This work was carried out with this objective, and is in logical continuity with previous studies using low-dose CT. These low-dose CT studies demonstrated no significant differences with the reference high dose CT in the depiction of parietal pleural fibrosis (*i*.*e*. pleural plaques) or parenchymal fibrosis [23], and detected 3 to 5 times more cases of pleural thickening and interstitial lung disease–the majority being minor–when compared to PA chest radiographs [24]. Low-dose CT examinations had also the ability to demonstrate parenchymal lung manifestations in a higher-risk asymptomatic sub-group of exposed patients [25], and showed a utility for lung cancer screening in this specific population [26].

However, these low-dose examinations performed between 2000 and 2010 had an effective dose estimated at 1.5–2 mSv. This dose, which was at the time considered to be "low", is 6 to 8 times higher than the Ultra-Low-Dose technique used in the present study, and almost corresponds to a standard dose nowadays [27], being only 2 times lower than the full dose acquisition recorded in our study. This illustrates well the technological leap taken by CT in the last ten years, driven by the introduction of Iterative Reconstructions. It allows a drastic reduction in the dose while maintaining image quality [8–10, 28, 29], by reducing background noise and a certain number of artefacts. For the acquisition settings of the ULD CT, we have chosen to decrease the tube current to 10 mA and to maintain a high voltage of 135 kV. We have decided to foster a “higher kV / low mA” technique rather than a "low kV / higher mA” such as 80 kV / 40 mA in order to reduce streak artifacts, improve image quality in the upper and lower areas of the lung and reduce the dose delivered to the superficial structures (skin, thyroid, breast), while being more robust to the image deterioration in obese patient [13].

Considering the diagnostic performance of the ULD CT in our study, it appears justified to suggest it as a first-line screening test, to be completed only in doubtful or positive cases by a full dose chest CT. If we consider a rough abnormalities prevalence of 20%, it means that 80% of patients could settle with only a ULD CT, whereas the remaining 20% would receive a full dose CT in addition to the initial ULD CT. In the end, such attitude would allow a reduction in the radiation exposure of the screening population of almost 75%. The major problem induced by this suggested screening method is organisational: to avoid recalling 20% of the patients, the radiologist must be present when the screening takes place in order to interpret the images and to determine if a full dose CT is required. This is possible in France for instance, where the radiologist is physically present during the examination, but is difficult to carry out in other countries such as North America where the radiologist is usually elsewhere during the CT acquisitions.

### Our study has some limitations

The most evident one is the limited size of the sample. 55 patients is indeed small, but has the advantage of being homogenous since all patients were managed in the same centre, and is two times higher than the population reported by Tekath et al. [16]. The prevalence of the different abnormalities in our study corresponds to those expected on a national scale [15]. In particular, asbestosis prevalence was 7.2% (4 out of 55 patients), which is on par with recent studies [30, 31]. Because of the small sample, we had chosen a non-inferiority threshold of 10%. This is open for discussion from a clinical perspective but was chosen at the beginning of the study in the hope of obtaining statistical significance. In order to reach a more classical non-inferiority threshold of 5% and based on a prevalence of abnormalities of 20%, the study would have had to include 140 patients, which would have required more than 2 years of inclusion. Consequently, due to the low prevalence of asbestosis, ULD sensitivity and PPV for this specific sign remains limited, however the specificity and the NPV remains over 90%, which is an essential feature for a first-line screening tool.

Another limitation that could be raised is that this work is vendor specific. Actually, and in order to harmonize our data, we decided to scan all our patients with the same machine and the same IR algorithm. Since the effects of all current-generations IR are analogous, we do believe that our results can be generalized. We have indeed witnessed it on our 64-row scanner (*CT750HD*, GE, Milwaukee, USA) equipped with another IR algorithm (*ASIR*–Adaptive Statistical Iterative Reconstruction), where image quality of ULD CT is visually similar to the one obtained in this study.

In conclusion, this study strengthens the diagnostic performance of the ULD CT in the detection of pleuropulmonary diseases related to asbestos exposure. With a sensitivity of 90.9% and a negative predictive value of 97.8%, ULD is efficient in this indication while exposing patients to a radiation dose 6 to 8 times lower than the previously-studied low-dose CT and 16 times lower than the full dose reference acquisition. It could therefore be proposed as a first-line screening test, completed only in doubtful or positive cases by a full dose CT acquisition.

## Supporting Information

S1 Table Diagnostic performance of the ULD CT in joint analysis. (XLSX)

S2 Table Intra-reader agreement. (XLSX)
